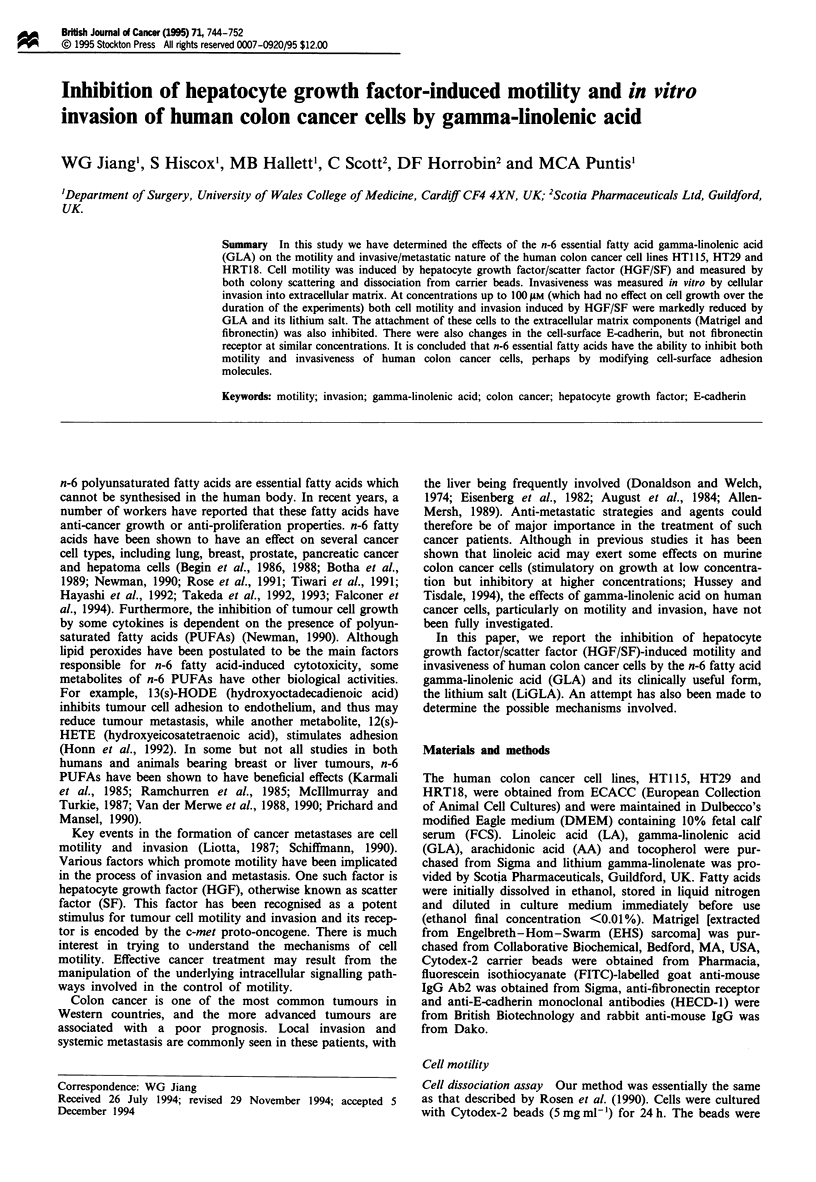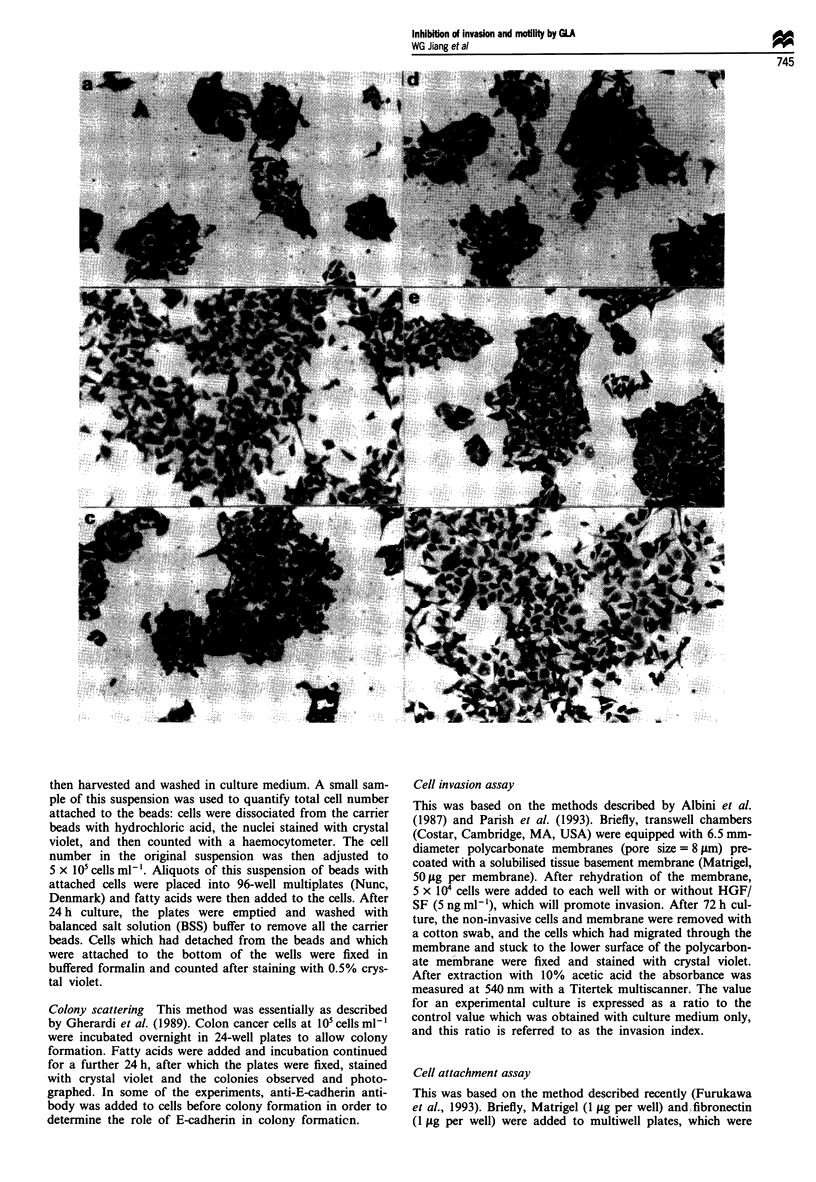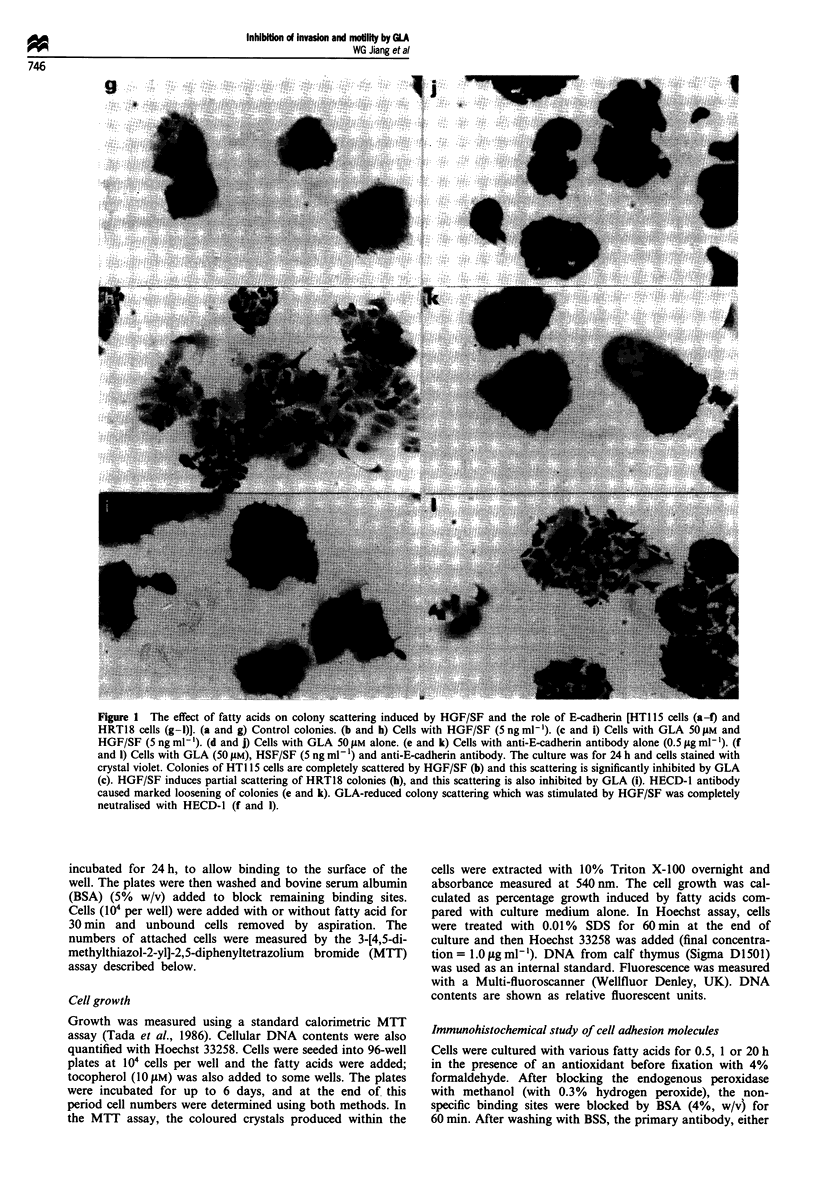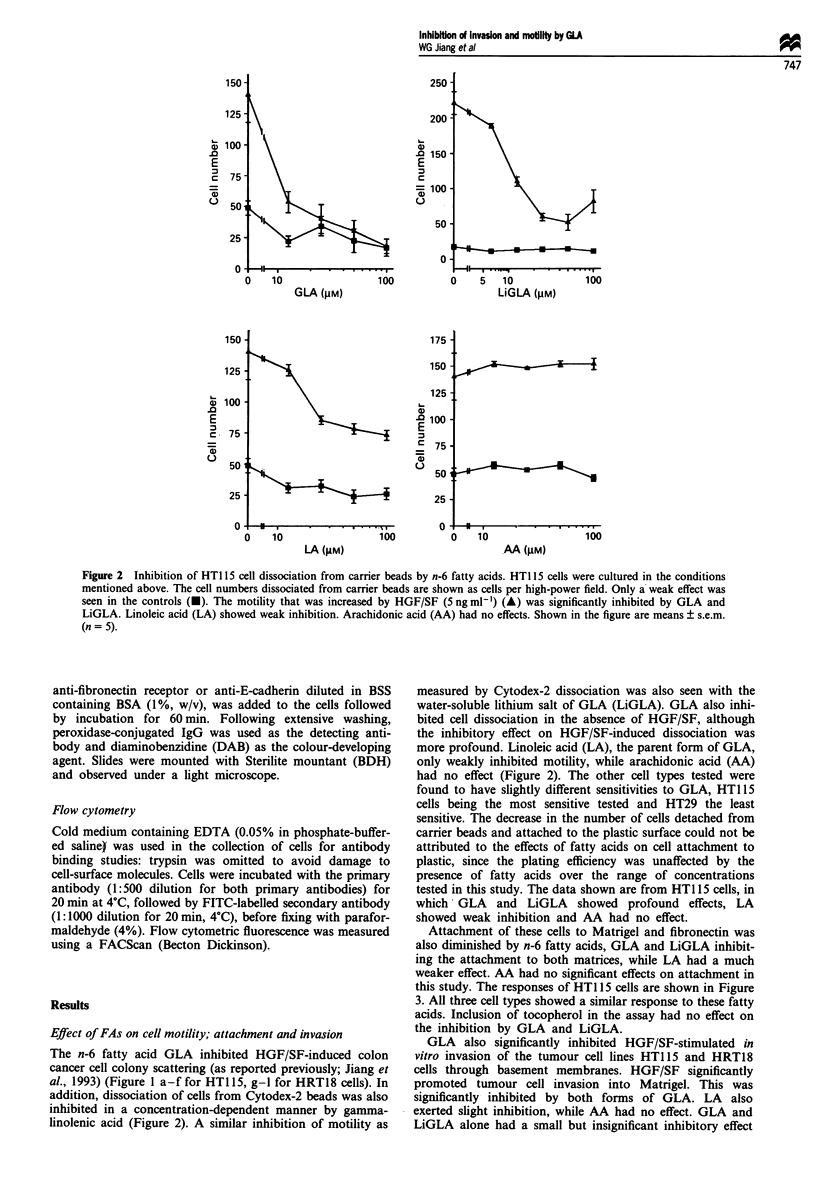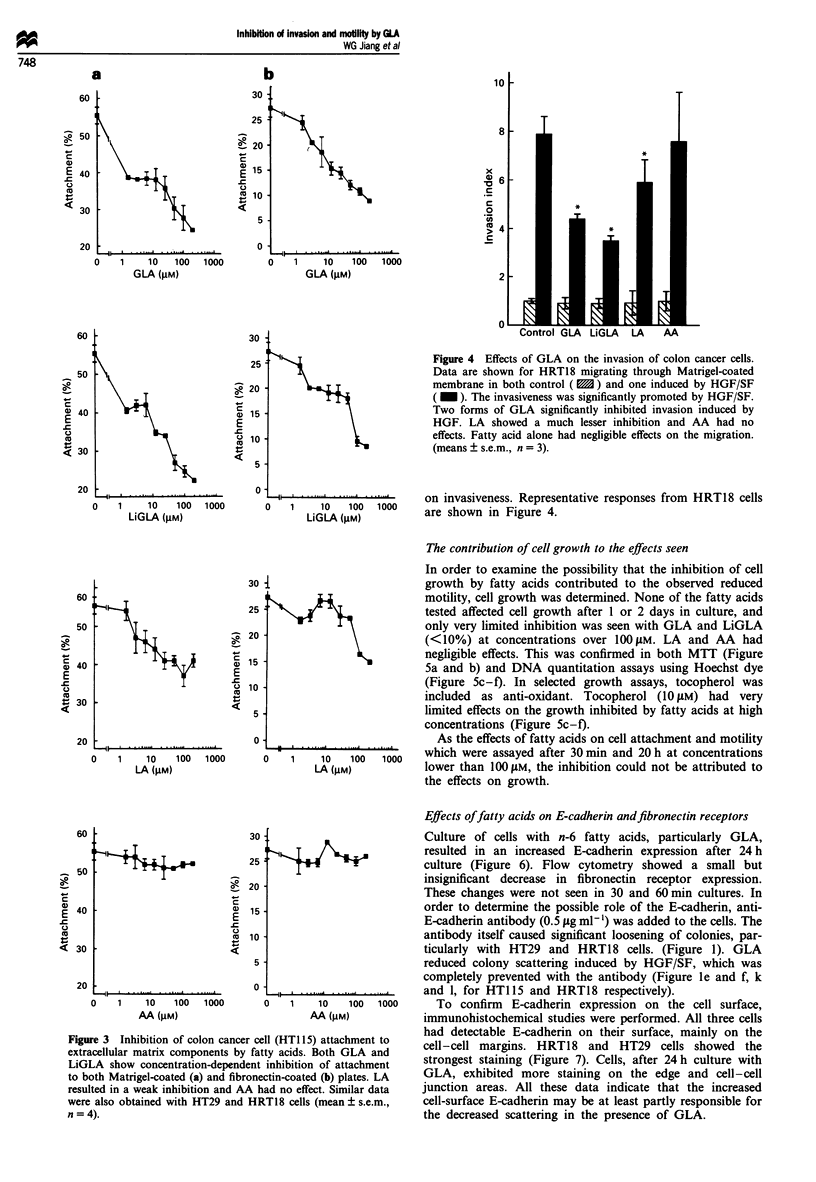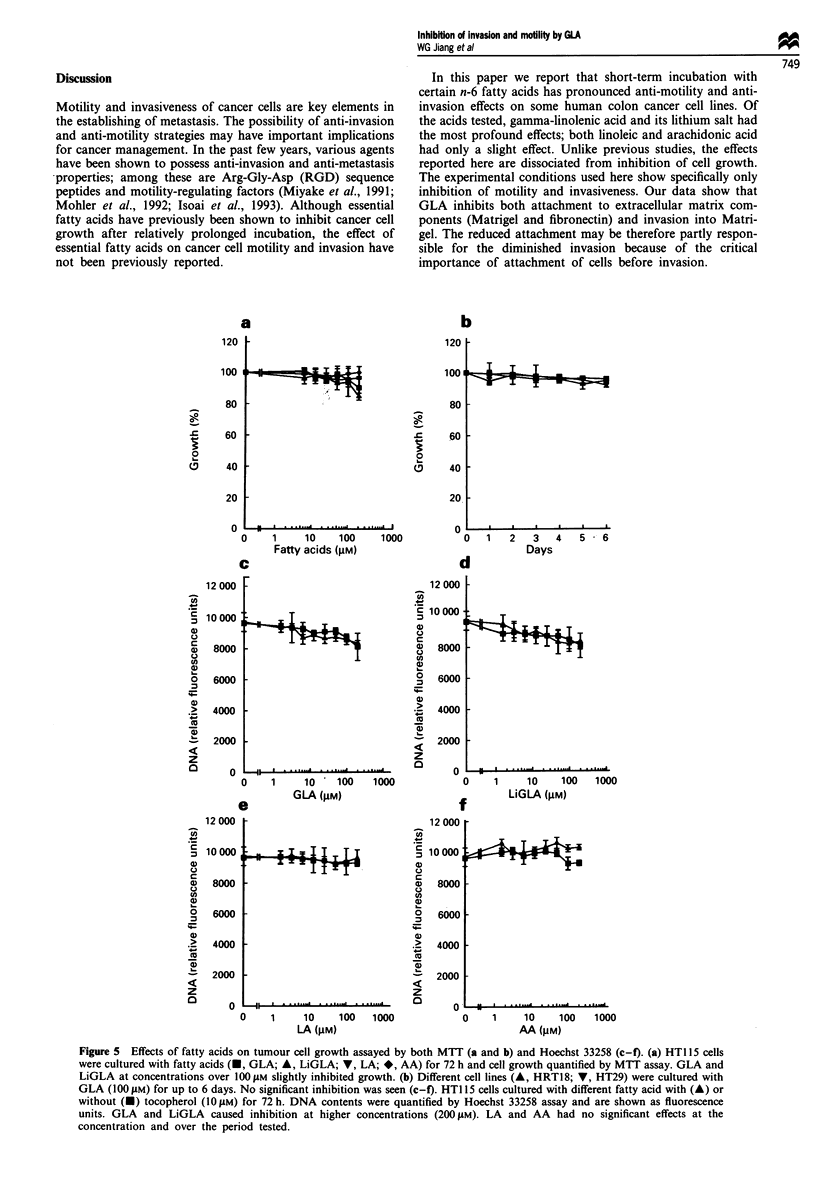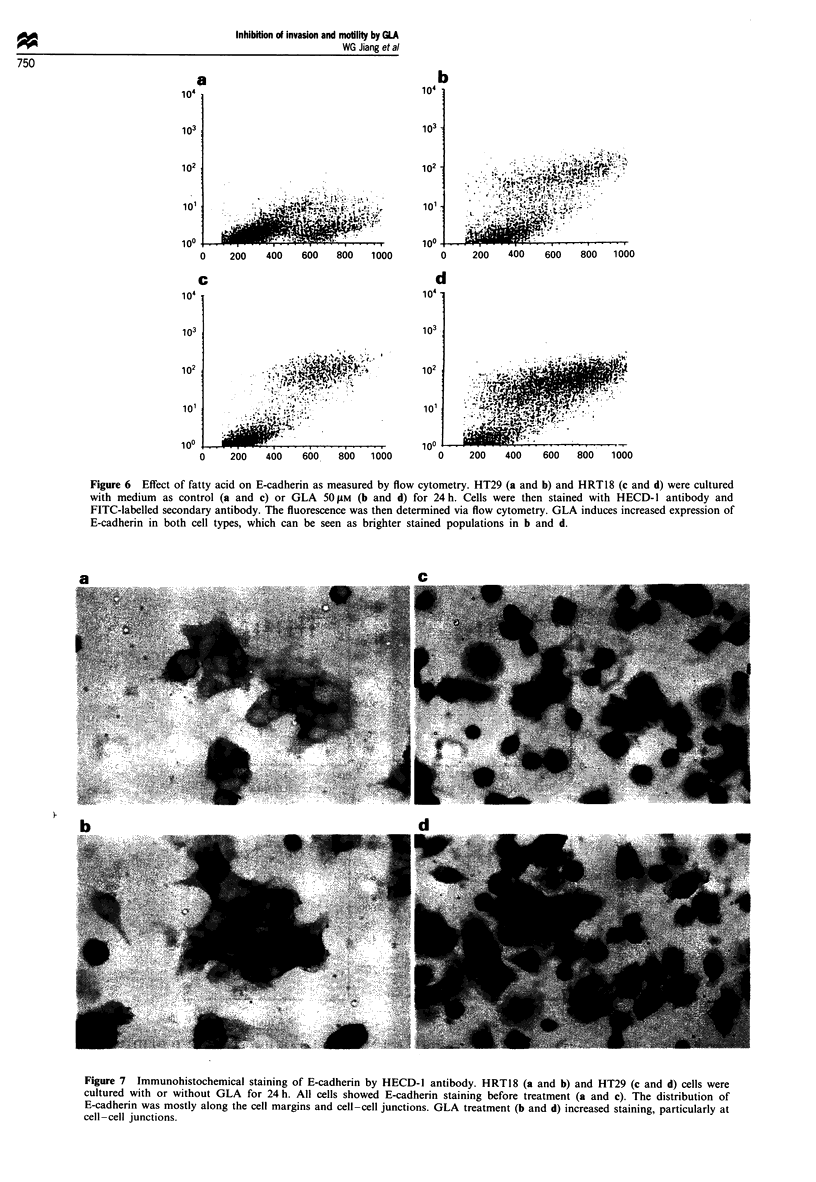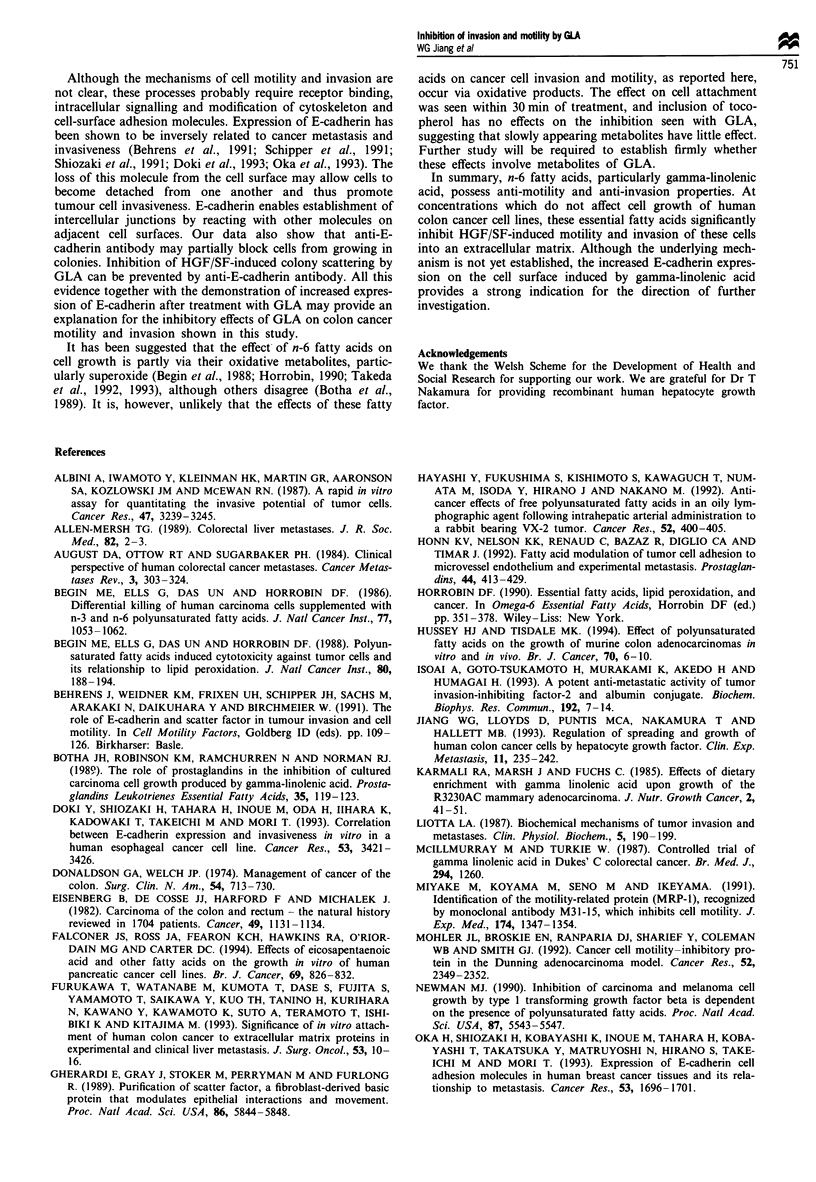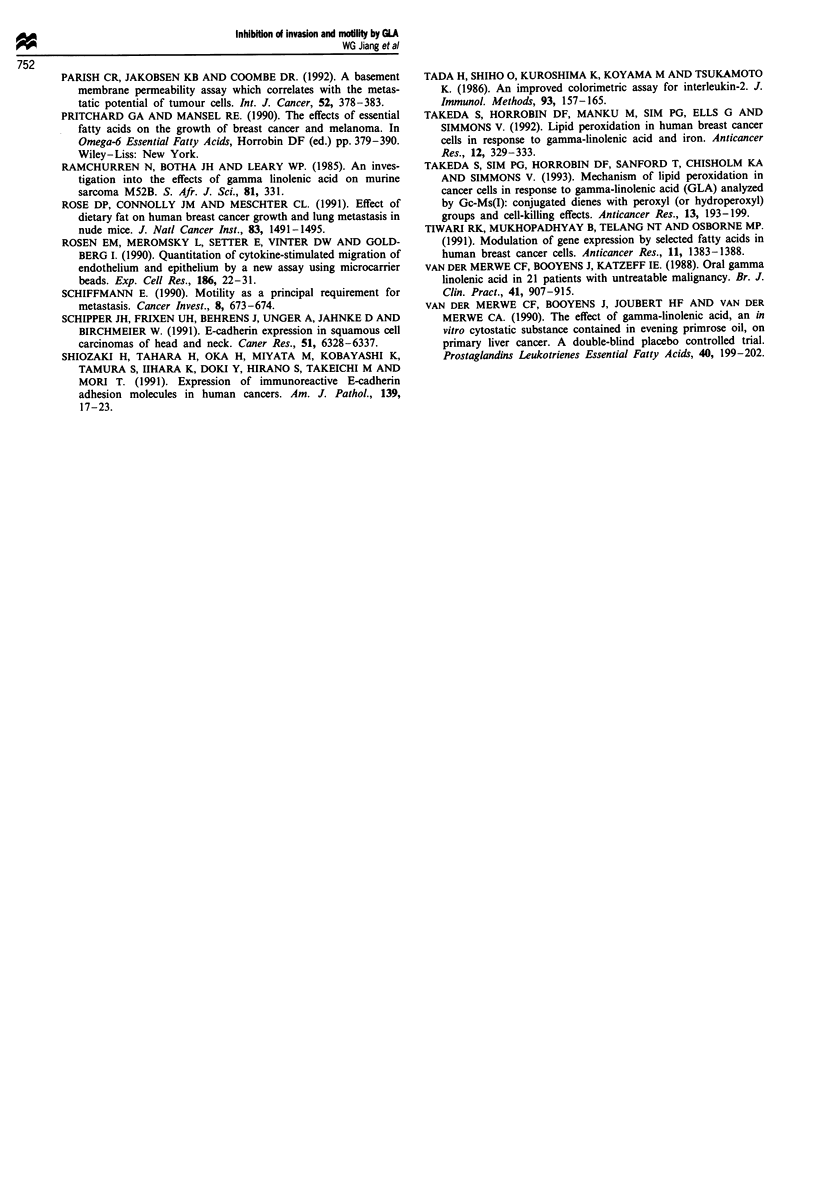# Inhibition of hepatocyte growth factor-induced motility and in vitro invasion of human colon cancer cells by gamma-linolenic acid.

**DOI:** 10.1038/bjc.1995.145

**Published:** 1995-04

**Authors:** W. G. Jiang, S. Hiscox, M. B. Hallett, C. Scott, D. F. Horrobin, M. C. Puntis

**Affiliations:** Department of Surgery, University of Wales College of Medicine, Cardiff, UK.

## Abstract

**Images:**


British Journal of Cancer (1995) 71, 744-752
© 1995 Stockton Press All rights reserved 0007-0920/95 $12.00

### Inhibition of hepatocyte growth factor-induced motility and *in vitro* invasion of human colon cancer cells by gamma-linolenic acid

WG Jiang[1], S Hiscox[1], MB Hallett[1], C Scott[2], DF Horrobin[2] and MCA Puntis[1]

[1]*Department of Surgery, University of Wales College of Medicine, Cardiff CF4 4XN, UK;* [2]*Scotia Pharmaceuticals Ltd, Guildford, UK.*

**Summary** In this study we have determined the effects of the *n*-6 essential fatty acid gamma-linolenic acid (GLA) on the motility and invasive/metastatic nature of the human colon cancer cell lines HT115, HT29 and HRT18. Cell motility was induced by hepatocyte growth factor/scatter factor (HGF/SF) and measured by both colony scattering and dissociation from carrier beads. Invasiveness was measured *in vitro* by cellular invasion into extracellular matrix. At concentrations up to 100 μM (which had no effect on cell growth over the duration of the experiments) both cell motility and invasion induced by HGF/SF were markedly reduced by GLA and its lithium salt. The attachment of these cells to the extracellular matrix components (Matrigel and fibronectin) was also inhibited. There were also changes in the cell-surface E-cadherin, but not fibronectin receptor at similar concentrations. It is concluded that *n*-6 essential fatty acids have the ability to inhibit both motility and invasiveness of human colon cancer cells, perhaps by modifying cell-surface adhesion molecules.

**Keywords:** motility; invasion; gamma-linolenic acid; colon cancer; hepatocyte growth factor; E-cadherin

*n*-6 polyunsaturated fatty acids are essential fatty acids which cannot be synthesised in the human body. In recent years, a number of workers have reported that these fatty acids have anti-cancer growth or anti-proliferation properties. *n*-6 fatty acids have been shown to have an effect on several cancer cell types, including lung, breast, prostate, pancreatic cancer and hepatoma cells (Begin *et al.*, 1986, 1988; Botha *et al.*, 1989; Newman, 1990; Rose *et al.*, 1991; Tiwari *et al.*, 1991; Hayashi *et al.*, 1992; Takeda *et al.*, 1992, 1993; Falconer *et al.*, 1994). Furthermore, the inhibition of tumour cell growth by some cytokines is dependent on the presence of polyunsaturated fatty acids (PUFAs) (Newman, 1990). Although lipid peroxides have been postulated to be the main factors responsible for *n*-6 fatty acid-induced cytotoxicity, some metabolites of *n*-6 PUFAs have other biological activities. For example, 13(s)-HODE (hydroxyoctadecadienoic acid) inhibits tumour cell adhesion to endothelium, and thus may reduce tumour metastasis, while another metabolite, 12(s)-HETE (hydroxyeicosatetraenoic acid), stimulates adhesion (Honn *et al.*, 1992). In some but not all studies in both humans and animals bearing breast or liver tumours, *n*-6 PUFAs have been shown to have beneficial effects (Karmali *et al.*, 1985; Ramchurren *et al.*, 1985; McIllmurray and Turkie, 1987; Van der Merwe *et al.*, 1988, 1990; Prichard and Mansel, 1990).

Key events in the formation of cancer metastases are cell motility and invasion (Liotta, 1987; Schiffmann, 1990). Various factors which promote motility have been implicated in the process of invasion and metastasis. One such factor is hepatocyte growth factor (HGF), otherwise known as scatter factor (SF). This factor has been recognised as a potent stimulus for tumour cell motility and invasion and its receptor is encoded by the c-*met* proto-oncogene. There is much interest in trying to understand the mechanisms of cell motility. Effective cancer treatment may result from the manipulation of the underlying intracellular signalling pathways involved in the control of motility.

Colon cancer is one of the most common tumours in Western countries, and the more advanced tumours are associated with a poor prognosis. Local invasion and systemic metastasis are commonly seen in these patients, with the liver being frequently involved (Donaldson and Welch, 1974; Eisenberg *et al.*, 1982; August *et al.*, 1984; Allen-Mersh, 1989). Anti-metastatic strategies and agents could therefore be of major importance in the treatment of such cancer patients. Although in previous studies it has been shown that linoleic acid may exert some effects on murine colon cancer cells (stimulatory on growth at low concentration but inhibitory at higher concentrations; Hussey and Tisdale, 1994), the effects of gamma-linolenic acid on human cancer cells, particularly on motility and invasion, have not been fully investigated.

In this paper, we report the inhibition of hepatocyte growth factor/scatter factor (HGF/SF)-induced motility and invasiveness of human colon cancer cells by the *n*-6 fatty acid gamma-linolenic acid (GLA) and its clinically useful form, the lithium salt (LiGLA). An attempt has also been made to determine the possible mechanisms involved.

### Materials and methods

The human colon cancer cell lines, HT115, HT29 and HRT18, were obtained from ECACC (European Collection of Animal Cell Cultures) and were maintained in Dulbecco's modified Eagle medium (DMEM) containing 10% fetal calf serum (FCS). Linoleic acid (LA), gamma-linolenic acid (GLA), arachidonic acid (AA) and tocopherol were purchased from Sigma and lithium gamma-linolenate was provided by Scotia Pharmaceuticals, Guildford, UK. Fatty acids were initially dissolved in ethanol, stored in liquid nitrogen and diluted in culture medium immediately before use (ethanol final concentration <0.01%). Matrigel [extracted from Engelbreth-Hom-Swarm (EHS) sarcoma] was purchased from Collaborative Biochemical, Bedford, MA, USA, Cytodex-2 carrier beads were obtained from Pharmacia, fluorescein isothiocyanate (FITC)-labelled goat anti-mouse IgG Ab2 was obtained from Sigma, anti-fibronectin receptor and anti-E-cadherin monoclonal antibodies (HECD-1) were from British Biotechnology and rabbit anti-mouse IgG was from Dako.

### *Cell motility*

*Cell dissociation assay* Our method was essentially the same as that described by Rosen *et al.* (1990). Cells were cultured with Cytodex-2 beads (5 mg $ml^{-1}$) for 24 h. The beads were

Correspondence: WG Jiang
Received 26 July 1994; revised 29 November 1994; accepted 5 December 1994

then harvested and washed in culture medium. A small sample of this suspension was used to quantify total cell number attached to the beads: cells were dissociated from the carrier beads with hydrochloric acid, the nuclei stained with crystal violet, and then counted with a haemocytometer. The cell number in the original suspension was then adjusted to $5 \times 10^5$ cells $ml^{-1}$. Aliquots of this suspension of beads with attached cells were placed into 96-well multiplates (Nunc, Denmark) and fatty acids were then added to the cells. After 24 h culture, the plates were emptied and washed with balanced salt solution (BSS) buffer to remove all the carrier beads. Cells which had detached from the beads and which were attached to the bottom of the wells were fixed in buffered formalin and counted after staining with 0.5% crystal violet.

*Colony scattering* This method was essentially as described by Gherardi *et al.* (1989). Colon cancer cells at $10^5$ cells $ml^{-1}$ were incubated overnight in 24-well plates to allow colony formation. Fatty acids were added and incubation continued for a further 24 h, after which the plates were fixed, stained with crystal violet and the colonies observed and photographed. In some of the experiments, anti-E-cadherin antibody was added to cells before colony formation in order to determine the role of E-cadherin in colony formation.

### *Cell invasion assay*

This was based on the methods described by Albini *et al.* (1987) and Parish *et al.* (1993). Briefly, transwell chambers (Costar, Cambridge, MA, USA) were equipped with 6.5 mm-diameter polycarbonate membranes (pore size = 8 μm) precoated with a solubilised tissue basement membrane (Matrigel, 50 μg per membrane). After rehydration of the membrane, $5 \times 10^4$ cells were added to each well with or without HGF/SF (5 ng $ml^{-1}$), which will promote invasion. After 72 h culture, the non-invasive cells and membrane were removed with a cotton swab, and the cells which had migrated through the membrane and stuck to the lower surface of the polycarbonate membrane were fixed and stained with crystal violet. After extraction with 10% acetic acid the absorbance was measured at 540 nm with a Titertek multiscanner. The value for an experimental culture is expressed as a ratio to the control value which was obtained with culture medium only, and this ratio is referred to as the invasion index.

### *Cell attachment assay*

This was based on the method described recently (Furukawa *et al.*, 1993). Briefly, Matrigel (1 μg per well) and fibronectin (1 μg per well) were added to multiwell plates, which were

**Figure 1** The effect of fatty acids on colony scattering induced by HGF/SF and the role of E-cadherin [HT115 cells (**a**–**f**) and HRT18 cells (**g**–**l**)]. (**a** and **g**) Control colonies. (**b** and **h**) Cells with HGF/SF (5 ng ml$^{-1}$). (**c** and **i**) Cells with GLA 50 μM and HGF/SF (5 ng ml$^{-1}$). (**d** and **j**) Cells with GLA 50 μM alone. (**e** and **k**) Cells with anti-E-cadherin antibody alone (0.5 μg ml$^{-1}$). (**f** and **l**) Cells with GLA (50 μM), HSF/SF (5 ng ml$^{-1}$) and anti-E-cadherin antibody. The culture was for 24 h and cells stained with crystal violet. Colonies of HT115 cells are completely scattered by HGF/SF (**b**) and this scattering is significantly inhibited by GLA (**c**). HGF/SF induces partial scattering of HRT18 colonies (**h**), and this scattering is also inhibited by GLA (**i**). HECD-1 antibody caused marked loosening of colonies (**e** and **k**). GLA-reduced colony scattering which was stimulated by HGF/SF was completely neutralised with HECD-1 (**f** and **l**).

incubated for 24 h, to allow binding to the surface of the well. The plates were then washed and bovine serum albumin (BSA) (5% w/v) added to block remaining binding sites. Cells ($10^4$ per well) were added with or without fatty acid for 30 min and unbound cells removed by aspiration. The numbers of attached cells were measured by the 3-[4,5-dimethylthiazol-2-yl]-2,5-diphenyltetrazolium bromide (MTT) assay described below.

### *Cell growth*

Growth was measured using a standard calorimetric MTT assay (Tada *et al.*, 1986). Cellular DNA contents were also quantified with Hoechst 33258. Cells were seeded into 96-well plates at $10^4$ cells per well and the fatty acids were added; tocopherol (10 μM) was also added to some wells. The plates were incubated for up to 6 days, and at the end of this period cell numbers were determined using both methods. In the MTT assay, the coloured crystals produced within the cells were extracted with 10% Triton X-100 overnight and absorbance measured at 540 nm. The cell growth was calculated as percentage growth induced by fatty acids compared with culture medium alone. In Hoechst assay, cells were treated with 0.01% SDS for 60 min at the end of culture and then Hoechst 33258 was added (final concentration = 1.0 μg ml$^{-1}$). DNA from calf thymus (Sigma D1501) was used as an internal standard. Fluorescence was measured with a Multi-fluoroscanner (Wellfluor Denley, UK). DNA contents are shown as relative fluorescent units.

### *Immunohistochemical study of cell adhesion molecules*

Cells were cultured with various fatty acids for 0.5, 1 or 20 h in the presence of an antioxidant before fixation with 4% formaldehyde. After blocking the endogenous peroxidase with methanol (with 0.3% hydrogen peroxide), the non-specific binding sites were blocked by BSA (4%, w/v) for 60 min. After washing with BSS, the primary antibody, either

**Figure 2** Inhibition of HT115 cell dissociation from carrier beads by *n*-6 fatty acids. HT115 cells were cultured in the conditions mentioned above. The cell numbers dissociated from carrier beads are shown as cells per high-power field. Only a weak effect was seen in the controls (■). The motility that was increased by HGF/SF (5 ng ml$^{-1}$) (▲) was significantly inhibited by GLA and LiGLA. Linoleic acid (LA) showed weak inhibition. Arachidonic acid (AA) had no effects. Shown in the figure are means ± s.e.m. ($n = 5$).

anti-fibronectin receptor or anti-E-cadherin diluted in BSS containing BSA (1%, w/v), was added to the cells followed by incubation for 60 min. Following extensive washing, peroxidase-conjugated IgG was used as the detecting antibody and diaminobenzidine (DAB) as the colour-developing agent. Slides were mounted with Sterilite mountant (BDH) and observed under a light microscope.

### *Flow cytometry*

Cold medium containing EDTA (0.05% in phosphate-buffered saline) was used in the collection of cells for antibody binding studies: trypsin was omitted to avoid damage to cell-surface molecules. Cells were incubated with the primary antibody (1:500 dilution for both primary antibodies) for 20 min at 4°C, followed by FITC-labelled secondary antibody (1:1000 dilution for 20 min, 4°C), before fixing with paraformaldehyde (4%). Flow cytometric fluorescence was measured using a FACScan (Becton Dickinson).

### Results

### *Effect of FAs on cell motility; attachment and invasion*

The *n*-6 fatty acid GLA inhibited HGF/SF-induced colon cancer cell colony scattering (as reported previously; Jiang *et al*., 1993) (Figure 1 a–f for HT115, g–l for HRT18 cells). In addition, dissociation of cells from Cytodex-2 beads was also inhibited in a concentration-dependent manner by gamma-linolenic acid (Figure 2). A similar inhibition of motility as measured by Cytodex-2 dissociation was also seen with the water-soluble lithium salt of GLA (LiGLA). GLA also inhibited cell dissociation in the absence of HGF/SF, although the inhibitory effect on HGF/SF-induced dissociation was more profound. Linoleic acid (LA), the parent form of GLA, only weakly inhibited motility, while arachidonic acid (AA) had no effect (Figure 2). The other cell types tested were found to have slightly different sensitivities to GLA, HT115 cells being the most sensitive tested and HT29 the least sensitive. The decrease in the number of cells detached from carrier beads and attached to the plastic surface could not be attributed to the effects of fatty acids on cell attachment to plastic, since the plating efficiency was unaffected by the presence of fatty acids over the range of concentrations tested in this study. The data shown are from HT115 cells, in which GLA and LiGLA showed profound effects, LA showed weak inhibition and AA had no effect.

Attachment of these cells to Matrigel and fibronectin was also diminished by *n*-6 fatty acids, GLA and LiGLA inhibiting the attachment to both matrices, while LA had a much weaker effect. AA had no significant effects on attachment in this study. The responses of HT115 cells are shown in Figure 3. All three cell types showed a similar response to these fatty acids. Inclusion of tocopherol in the assay had no effect on the inhibition by GLA and LiGLA.

GLA also significantly inhibited HGF/SF-stimulated *in vitro* invasion of the tumour cell lines HT115 and HRT18 cells through basement membranes. HGF/SF significantly promoted tumour cell invasion into Matrigel. This was significantly inhibited by both forms of GLA. LA also exerted slight inhibition, while AA had no effect. GLA and LiGLA alone had a small but insignificant inhibitory effect

**Figure 3** Inhibition of colon cancer cell (HT115) attachment to extracellular matrix components by fatty acids. Both GLA and LiGLA show concentration-dependent inhibition of attachment to both Matrigel-coated (**a**) and fibronectin-coated (**b**) plates. LA resulted in a weak inhibition and AA had no effect. Similar data were also obtained with HT29 and HRT18 cells (mean ± s.e.m., $n = 4$).

**Figure 4** Effects of GLA on the invasion of colon cancer cells. Data are shown for HRT18 migrating through Matrigel-coated membrane in both control (▨) and one induced by HGF/SF (■). The invasiveness was significantly promoted by HGF/SF. Two forms of GLA significantly inhibited invasion induced by HGF. LA showed a much lesser inhibition and AA had no effects. Fatty acid alone had negligible effects on the migration. (means ± s.e.m., $n = 3$).

on invasiveness. Representative responses from HRT18 cells are shown in Figure 4.

*The contribution of cell growth to the effects seen*

In order to examine the possibility that the inhibition of cell growth by fatty acids contributed to the observed reduced motility, cell growth was determined. None of the fatty acids tested affected cell growth after 1 or 2 days in culture, and only very limited inhibition was seen with GLA and LiGLA (<10%) at concentrations over 100 μM. LA and AA had negligible effects. This was confirmed in both MTT (Figure 5a and b) and DNA quantitation assays using Hoechst dye (Figure 5c–f). In selected growth assays, tocopherol was included as anti-oxidant. Tocopherol (10 μM) had very limited effects on the growth inhibited by fatty acids at high concentrations (Figure 5c–f).

As the effects of fatty acids on cell attachment and motility which were assayed after 30 min and 20 h at concentrations lower than 100 μM, the inhibition could not be attributed to the effects on growth.

*Effects of fatty acids on E-cadherin and fibronectin receptors*

Culture of cells with *n*-6 fatty acids, particularly GLA, resulted in an increased E-cadherin expression after 24 h culture (Figure 6). Flow cytometry showed a small but insignificant decrease in fibronectin receptor expression. These changes were not seen in 30 and 60 min cultures. In order to determine the possible role of the E-cadherin, anti-E-cadherin antibody (0.5 μg $ml^{-1}$) was added to the cells. The antibody itself caused significant loosening of colonies, particularly with HT29 and HRT18 cells. (Figure 1). GLA reduced colony scattering induced by HGF/SF, which was completely prevented with the antibody (Figure 1e and f, k and l, for HT115 and HRT18 respectively).

To confirm E-cadherin expression on the cell surface, immunohistochemical studies were performed. All three cells had detectable E-cadherin on their surface, mainly on the cell–cell margins. HRT18 and HT29 cells showed the strongest staining (Figure 7). Cells, after 24 h culture with GLA, exhibited more staining on the edge and cell–cell junction areas. All these data indicate that the increased cell-surface E-cadherin may be at least partly responsible for the decreased scattering in the presence of GLA.

### Discussion

Motility and invasiveness of cancer cells are key elements in the establishing of metastasis. The possibility of anti-invasion and anti-motility strategies may have important implications for cancer management. In the past few years, various agents have been shown to possess anti-invasion and anti-metastasis properties; among these are Arg-Gly-Asp (RGD) sequence peptides and motility-regulating factors (Miyake et al., 1991; Mohler et al., 1992; Isoai et al., 1993). Although essential fatty acids have previously been shown to inhibit cancer cell growth after relatively prolonged incubation, the effect of essential fatty acids on cancer cell motility and invasion have not been previously reported.

In this paper we report that short-term incubation with certain n-6 fatty acids has pronounced anti-motility and anti-invasion effects on some human colon cancer cell lines. Of the acids tested, gamma-linolenic acid and its lithium salt had the most profound effects; both linoleic and arachidonic acid had only a slight effect. Unlike previous studies, the effects reported here are dissociated from inhibition of cell growth. The experimental conditions used here show specifically only inhibition of motility and invasiveness. Our data show that GLA inhibits both attachment to extracellular matrix components (Matrigel and fibronectin) and invasion into Matrigel. The reduced attachment may be therefore partly responsible for the diminished invasion because of the critical importance of attachment of cells before invasion.

**Figure 5** Effects of fatty acids on tumour cell growth assayed by both MTT (a and b) and Hoechst 33258 (c–f). (a) HT115 cells were cultured with fatty acids (■, GLA; ▲, LiGLA; ▼, LA; ◆, AA) for 72 h and cell growth quantified by MTT assay. GLA and LiGLA at concentrations over 100 μM slightly inhibited growth. (b) Different cell lines (▲, HRT18; ▼, HT29) were cultured with GLA (100 μM) for up to 6 days. No significant inhibition was seen (c–f). HT115 cells cultured with different fatty acid with (▲) or without (■) tocopherol (10 μM) for 72 h. DNA contents were quantified by Hoechst 33258 assay and are shown as fluorescence units. GLA and LiGLA caused inhibition at higher concentrations (200 μM). LA and AA had no significant effects at the concentration and over the period tested.

**Figure 6** Effect of fatty acid on E-cadherin as measured by flow cytometry. HT29 (**a** and **b**) and HRT18 (**c** and **d**) were cultured with medium as control (**a** and **c**) or GLA 50 μM (**b** and **d**) for 24 h. Cells were then stained with HECD-1 antibody and FITC-labelled secondary antibody. The fluorescence was then determined via flow cytometry. GLA induces increased expression of E-cadherin in both cell types, which can be seen as brighter stained populations in **b** and **d**.

**Figure 7** Immunohistochemical staining of E-cadherin by HECD-1 antibody. HRT18 (**a** and **b**) and HT29 (**c** and **d**) cells were cultured with or without GLA for 24 h. All cells showed E-cadherin staining before treatment (**a** and **c**). The distribution of E-cadherin was mostly along the cell margins and cell–cell junctions. GLA treatment (**b** and **d**) increased staining, particularly at cell–cell junctions.

Although the mechanisms of cell motility and invasion are not clear, these processes probably require receptor binding, intracellular signalling and modification of cytoskeleton and cell-surface adhesion molecules. Expression of E-cadherin has been shown to be inversely related to cancer metastasis and invasiveness (Behrens *et al.*, 1991; Schipper *et al.*, 1991; Shiozaki *et al.*, 1991; Doki *et al.*, 1993; Oka *et al.*, 1993). The loss of this molecule from the cell surface may allow cells to become detached from one another and thus promote tumour cell invasiveness. E-cadherin enables establishment of intercellular junctions by reacting with other molecules on adjacent cell surfaces. Our data also show that anti-E-cadherin antibody may partially block cells from growing in colonies. Inhibition of HGF/SF-induced colony scattering by GLA can be prevented by anti-E-cadherin antibody. All this evidence together with the demonstration of increased expression of E-cadherin after treatment with GLA may provide an explanation for the inhibitory effects of GLA on colon cancer motility and invasion shown in this study.

It has been suggested that the effect of *n*-6 fatty acids on cell growth is partly via their oxidative metabolites, particularly superoxide (Begin *et al.*, 1988; Horrobin, 1990; Takeda *et al.*, 1992, 1993), although others disagree (Botha *et al.*, 1989). It is, however, unlikely that the effects of these fatty acids on cancer cell invasion and motility, as reported here, occur via oxidative products. The effect on cell attachment was seen within 30 min of treatment, and inclusion of tocopherol has no effects on the inhibition seen with GLA, suggesting that slowly appearing metabolites have little effect. Further study will be required to establish firmly whether these effects involve metabolites of GLA.

In summary, *n*-6 fatty acids, particularly gamma-linolenic acid, possess anti-motility and anti-invasion properties. At concentrations which do not affect cell growth of human colon cancer cell lines, these essential fatty acids significantly inhibit HGF/SF-induced motility and invasion of these cells into an extracellular matrix. Although the underlying mechanism is not yet established, the increased E-cadherin expression on the cell surface induced by gamma-linolenic acid provides a strong indication for the direction of further investigation.

**Acknowledgements**
We thank the Welsh Scheme for the Development of Health and Social Research for supporting our work. We are grateful for Dr T Nakamura for providing recombinant human hepatocyte growth factor.

### References

ALBINI A, IWAMOTO Y, KLEINMAN HK, MARTIN GR, AARONSON SA, KOZLOWSKI JM AND MCEWAN RN. (1987). A rapid *in vitro* assay for quantitating the invasive potential of tumor cells. *Cancer Res.*, **47**, 3239–3245.

ALLEN-MERSH TG. (1989). Colorectal liver metastases. *J. R. Soc. Med.*, **82**, 2–3.

AUGUST DA, OTTOW RT AND SUGARBAKER PH. (1984). Clinical perspective of human colorectal cancer metastases. *Cancer Metastases Rev.*, **3**, 303–324.

BEGIN ME, ELLS G, DAS UN AND HORROBIN DF. (1986). Differential killing of human carcinoma cells supplemented with n-3 and n-6 polyunsaturated fatty acids. *J. Natl Cancer Inst.*, **77**, 1053–1062.

BEGIN ME, ELLS G, DAS UN AND HORROBIN DF. (1988). Polyunsaturated fatty acids induced cytotoxicity against tumor cells and its relationship to lipid peroxidation. *J. Natl Cancer Inst.*, **80**, 188–194.

BEHRENS J, WEIDNER KM, FRIXEN UH, SCHIPPER JH, SACHS M, ARAKAKI N, DAIKUHARA Y AND BIRCHMEIER W. (1991). The role of E-cadherin and scatter factor in tumour invasion and cell motility. In *Cell Motility Factors*, Goldberg ID (eds). pp. 109–126. Birkharser: Basle.

BOTHA JH, ROBINSON KM, RAMCHURREN N AND NORMAN RJ. (1989). The role of prostaglandins in the inhibition of cultured carcinoma cell growth produced by gamma-linolenic acid. *Prostaglandins Leukotrienes Essential Fatty Acids*, **35**, 119–123.

DOKI Y, SHIOZAKI H, TAHARA H, INOUE M, ODA H, IIHARA K, KADOWAKI T, TAKEICHI M AND MORI T. (1993). Correlation between E-cadherin expression and invasiveness *in vitro* in a human esophageal cancer cell line. *Cancer Res.*, **53**, 3421–3426.

DONALDSON GA, WELCH JP. (1974). Management of cancer of the colon. *Surg. Clin. N. Am.*, **54**, 713–730.

EISENBERG B, DE COSSE JJ, HARFORD F AND MICHALEK J. (1982). Carcinoma of the colon and rectum – the natural history reviewed in 1704 patients. *Cancer*, **49**, 1131–1134.

FALCONER JS, ROSS JA, FEARON KCH, HAWKINS RA, O'RIORDAIN MG AND CARTER DC. (1994). Effects of eicosapentaenoic acid and other fatty acids on the growth *in vitro* of human pancreatic cancer cell lines. *Br. J. Cancer*, **69**, 826–832.

FURUKAWA T, WATANABE M, KUMOTA T, DASE S, FUJITA S, YAMAMOTO T, SAIKAWA Y, KUO TH, TANINO H, KURIHARA N, KAWANO Y, KAWAMOTO K, SUTO A, TERAMOTO T, ISHIBIKI K AND KITAJIMA M. (1993). Significance of *in vitro* attachment of human colon cancer to extracellular matrix proteins in experimental and clinical liver metastasis. *J. Surg. Oncol.*, **53**, 10–16.

GHERARDI E, GRAY J, STOKER M, PERRYMAN M AND FURLONG R. (1989). Purification of scatter factor, a fibroblast-derived basic protein that modulates epithelial interactions and movement. *Proc. Natl Acad. Sci. USA*, **86**, 5844–5848.

HAYASHI Y, FUKUSHIMA S, KISHIMOTO S, KAWAGUCH T, NUMATA M, ISODA Y, HIRANO J AND NAKANO M. (1992). Anticancer effects of free polyunsaturated fatty acids in an oily lymphographic agent following intrahepatic arterial administration to a rabbit bearing VX-2 tumor. *Cancer Res.*, **52**, 400–405.

HONN KV, NELSON KK, RENAUD C, BAZAZ R, DIGLIO CA AND TIMAR J. (1992). Fatty acid modulation of tumor cell adhesion to microvessel endothelium and experimental metastasis. *Prostaglandins*, **44**, 413–429.

HORROBIN DF. (1990). Essential fatty acids, lipid peroxidation, and cancer. In *Omega-6 Essential Fatty Acids*, Horrobin DF (ed.) pp. 351–378. Wiley–Liss: New York.

HUSSEY HJ AND TISDALE MK. (1994). Effect of polyunsaturated fatty acids on the growth of murine colon adenocarcinomas *in vitro* and *in vivo*. *Br. J. Cancer*, **70**, 6–10.

ISOAI A, GOTO-TSUKAMOTO H, MURAKAMI K, AKEDO H AND HUMAGAI H. (1993). A potent anti-metastatic activity of tumor invasion-inhibiting factor-2 and albumin conjugate. *Biochem. Biophys. Res. Commun.*, **192**, 7–14.

JIANG WG, LLOYDS D, PUNTIS MCA, NAKAMURA T AND HALLETT MB. (1993). Regulation of spreading and growth of human colon cancer cells by hepatocyte growth factor. *Clin. Exp. Metastasis*, **11**, 235–242.

KARMALI RA, MARSH J AND FUCHS C. (1985). Effects of dietary enrichment with gamma linolenic acid upon growth of the R3230AC mammary adenocarcinoma. *J. Nutr. Growth Cancer*, **2**, 41–51.

LIOTTA LA. (1987). Biochemical mechanisms of tumor invasion and metastases. *Clin. Physiol. Biochem.*, **5**, 190–199.

MCILLMURRAY M AND TURKIE W. (1987). Controlled trial of gamma linolenic acid in Dukes' C colorectal cancer. *Br. Med. J.*, **294**, 1260.

MIYAKE M, KOYAMA M, SENO M AND IKEYAMA. (1991). Identification of the motility-related protein (MRP-1), recognized by monoclonal antibody M31-15, which inhibits cell motility. *J. Exp. Med.*, **174**, 1347–1354.

MOHLER JL, BROSKIE EN, RANPARIA DJ, SHARIEF Y, COLEMAN WB AND SMITH GJ. (1992). Cancer cell motility–inhibitory protein in the Dunning adenocarcinoma model. *Cancer Res.*, **52**, 2349–2352.

NEWMAN MJ. (1990). Inhibition of carcinoma and melanoma cell growth by type 1 transforming growth factor beta is dependent on the presence of polyunsaturated fatty acids. *Proc. Natl Acad. Sci. USA*, **87**, 5543–5547.

OKA H, SHIOZAKI H, KOBAYASHI K, INOUE M, TAHARA H, KOBAYASHI T, TAKATSUKA Y, MATRUYOSHI N, HIRANO S, TAKEICHI M AND MORI T. (1993). Expression of E-cadherin cell adhesion molecules in human breast cancer tissues and its relationship to metastasis. *Cancer Res.*, **53**, 1696–1701.

PARISH CR, JAKOBSEN KB AND COOMBE DR. (1992). A basement membrane permeability assay which correlates with the metastatic potential of tumour cells. *Int. J. Cancer*, **52**, 378–383.

PRITCHARD GA AND MANSEL RE. (1990). The effects of essential fatty acids on the growth of breast cancer and melanoma. In *Omega-6 Essential Fatty Acids*, Horrobin DF (ed.) pp. 379–390. Wiley–Liss: New York.

RAMCHURREN N, BOTHA JH AND LEARY WP. (1985). An investigation into the effects of gamma linolenic acid on murine sarcoma M52B. *S. Afr. J. Sci.*, **81**, 331.

ROSE DP, CONNOLLY JM AND MESCHTER CL. (1991). Effect of dietary fat on human breast cancer growth and lung metastasis in nude mice. *J. Natl Cancer Inst.*, **83**, 1491–1495.

ROSEN EM, MEROMSKY L, SETTER E, VINTER DW AND GOLDBERG I. (1990). Quantitation of cytokine-stimulated migration of endothelium and epithelium by a new assay using microcarrier beads. *Exp. Cell Res.*, **186**, 22–31.

SCHIFFMANN E. (1990). Motility as a principal requirement for metastasis. *Cancer Invest.*, **8**, 673–674.

SCHIPPER JH, FRIXEN UH, BEHRENS J, UNGER A, JAHNKE D AND BIRCHMEIER W. (1991). E-cadherin expression in squamous cell carcinomas of head and neck. *Caner Res.*, **51**, 6328–6337.

SHIOZAKI H, TAHARA H, OKA H, MIYATA M, KOBAYASHI K, TAMURA S, IIHARA K, DOKI Y, HIRANO S, TAKEICHI M AND MORI T. (1991). Expression of immunoreactive E-cadherin adhesion molecules in human cancers. *Am. J. Pathol.*, **139**, 17–23.

TADA H, SHIHO O, KUROSHIMA K, KOYAMA M AND TSUKAMOTO K. (1986). An improved colorimetric assay for interleukin-2. *J. Immunol. Methods*, **93**, 157–165.

TAKEDA S, HORROBIN DF, MANKU M, SIM PG, ELLS G AND SIMMONS V. (1992). Lipid peroxidation in human breast cancer cells in response to gamma-linolenic acid and iron. *Anticancer Res.*, **12**, 329–333.

TAKEDA S, SIM PG, HORROBIN DF, SANFORD T, CHISHOLM KA AND SIMMONS V. (1993). Mechanism of lipid peroxidation in cancer cells in response to gamma-linolenic acid (GLA) analyzed by Gc-Ms(I): conjugated dienes with peroxyl (or hydroperoxyl) groups and cell-killing effects. *Anticancer Res.*, **13**, 193–199.

TIWARI RK, MUKHOPADHYAY B, TELANG NT AND OSBORNE MP. (1991). Modulation of gene expression by selected fatty acids in human breast cancer cells. *Anticancer Res.*, **11**, 1383–1388.

VAN DER MERWE CF, BOOYENS J, KATZEFF IE. (1988). Oral gamma linolenic acid in 21 patients with untreatable malignancy. *Br. J. Clin. Pract.*, **41**, 907–915.

VAN DER MERWE CF, BOOYENS J, JOUBERT HF AND VAN DER MERWE CA. (1990). The effect of gamma-linolenic acid, an *in vitro* cytostatic substance contained in evening primrose oil, on primary liver cancer. A double-blind placebo controlled trial. *Prostaglandins Leukotrienes Essential Fatty Acids*, **40**, 199–202.